# The Anti-Inflammatory and Antioxidant Properties of Acebuche Oil Exert a Retinoprotective Effect in a Murine Model of High-Tension Glaucoma

**DOI:** 10.3390/nu16030409

**Published:** 2024-01-30

**Authors:** Martina Lucchesi, Silvia Marracci, Rosario Amato, Dominga Lapi, Álvaro Santana-Garrido, Pablo Espinosa-Martín, Carmen María Vázquez, Alfonso Mate, Massimo Dal Monte

**Affiliations:** 1 Department of Biology, University of Pisa, 56127 Pisa, Italy; martina.lucchesi@med.unipi.it (M.L.); silvia.marracci@unipi.it (S.M.); rosario.amato@unipi.it (R.A.); dominga.lapi@unipi.it (D.L.); 2 Departamento de Fisiología, Facultad de Farmacia, Universidad de Sevilla, 41012 Sevilla, Spain; asgarrido@us.es (Á.S.-G.); pemartin@us.es (P.E.-M.); vazquez@us.es (C.M.V.); 3 Epidemiología Clínica y Riesgo Cardiovascular, Instituto de Biomedicina de Sevilla (IBIS), Hospital Universitario Virgen del Rocío/Consejo Superior de Investigaciones Científicas/Universidad de Sevilla, 41013 Sevilla, Spain; 4 Interdepartmental Research Center Nutrafood “Nutraceuticals and Food for Health”, University of Pisa, 56124 Pisa, Italy

**Keywords:** functional food, intraocular pressure, retinal function, electroretinography, neuroprotection, resilience

## Abstract

Glaucoma is characterized by cupping of the optic disc, apoptotic degeneration of retinal ganglion cells (RGCs) and their axons, and thinning of the retinal nerve fiber layer, with patchy loss of vision. Elevated intraocular pressure (IOP) is a major risk factor for hypertensive glaucoma and the only modifiable one. There is a need to find novel compounds that counteract other risk factors contributing to RGC degeneration. The oil derived from the wild olive tree (*Olea europaea* var. *sylvestris*), also called Acebuche (ACE), shows powerful anti-inflammatory, antioxidant and retinoprotective effects. We evaluated whether ACE oil could counteract glaucoma-related detrimental effects. To this aim, we fed mice either a regular or an ACE oil-enriched diet and then induced IOP elevation through intraocular injection of methylcellulose. An ACE oil-enriched diet suppressed glaucoma-dependent retinal glia reactivity and inflammation. The redox status of the glaucomatous retinas was restored to a control-like situation, and ischemia was alleviated by an ACE oil-enriched diet. Notably, retinal apoptosis was suppressed in the glaucomatous animals fed ACE oil. Furthermore, as shown by electroretinogram analyses, RGC electrophysiological functions were almost completely preserved by the ACE oil-enriched diet. These ameliorative effects were IOP-independent and might depend on ACE oil’s peculiar composition. Although additional studies are needed, nutritional supplementation with ACE oil might represent an adjuvant in the management of glaucoma.

## 1. Introduction

The term glaucoma refers to a group of lifelong progressive optic neuropathies characterized by excavation or cupping of the optic disc, apoptotic degeneration of retinal ganglion cells (RGCs) and their axons and thinning of the retinal nerve fiber layer. The optic nerve damage results in patchy loss of vision, usually starting in the periphery of the visual field; central vision is also progressively affected, and narrowing of vision is a symptom that can occur in both eyes. Individuals suffering from glaucoma may also complain of ocular pain and acute visual loss; however, glaucoma can also progress unnoticed by the patient until central visual acuity is affected [1]. Glaucoma is a major public health problem since it is the leading cause of irreversible blindness worldwide [2] and the second most common cause of blindness globally, after cataracts [3]. It is currently considered an age-dependent disease with pathogenetic mechanisms common to those of other neurodegenerative pathologies of the elderly; because of the growing proportion of older persons, it is predicted that more than 110 million people worldwide will have glaucoma by 2040 [4].

Elevated intraocular pressure (IOP), whose physiological levels range from 11 to 21 mmHg in humans [5], is considered a major risk factor for hypertensive glaucoma [6]. Physiological IOP is necessary to maintain the proper shape and optical properties of the eye globe [7]. Circadian rhythms and episcleral vein pressure contribute to determining its level, but the main player affecting IOP is the balance between production and drainage of aqueous humor [8]. The aqueous humor is a transparent fluid that fills and helps form the chambers of the eye. It is produced by the processes of the ciliary body in the posterior chamber, then flows around the lens and through the pupil into the anterior chamber and leaves the eye via both conventional and non-conventional pathways. Following the conventional route, the aqueous humor passes through the trabecular meshwork, across Schlemm’s canal, and into draining collector channels, such as the aqueous veins and the episcleral veins. The non-conventional pathway, instead, is represented by the uveal meshwork and the anterior face of the ciliary muscle. Disruption of aqueous humor outflow, which leads to increased IOP, usually occurs through the conventional route [9]. 

On the other hand, a large part of the patients showing cupping of the optic disc, RGC degeneration and thinning of the retinal nerve fiber layer have no statistically elevated IOP, a condition known as normal tension glaucoma, indicating that additional pathophysiological pathways other than IOP elevation may be involved in generating the damage characterizing glaucoma [10]. Among these IOP-independent mechanisms, insufficient vascular perfusion of the optic nerve head, dysregulation in the balance between apoptosis and autophagy in RGCs, oxidative stress and impaired neurotrophin production have been reported to play a fundamental role [11].

Although IOP is currently the solely modifiable risk factor for glaucoma and different medications are available to control it [12,13], it would be invaluable to find novel compounds to counteract other risk factors that contribute to RGC degeneration, especially for the often-observed situation of neurodegenerative progression in patients with pharmacologically controlled IOP [14,15], as well as for treating patients suffering from normotensive glaucoma [16]. In this respect, the use of neuroprotective treatments in clinical trials has not been fruitful so far [17] and, moreover, their scarce tolerability poses several concerns about their usage in clinics [18]. On the other hand, the possibility of using nutritional intervention to protect eyes and vision is increasing interest both at the preclinical and clinical levels, especially for those nutritional supplements able to prevent RGC degeneration, supporting and promoting RGC resilience to stress conditions [19,20]. For instance, two different epidemiological studies found that high rates of consumption of green leaves, vegetables, fruits and fruit juices reduced the risk of developing glaucoma [21,22]. Moreover, an antioxidant-enriched diet efficiently counteracted human glaucomatous-related pathologies by increasing the rate of blood circulation to the optic nerve and promoting RGC survival [23]. 

In this context, the wild olive tree (*Olea europaea* var. *sylvestris*), also called Acebuche (ACE), deserves attention. ACE olive tree is one of the oldest trees worldwide [24] and the oil derived from it contains tocopherols (e.g., vitamin E), sterols, triterpene acids and secoiridoid compounds in higher quantity compared to the classic extra virgin olive oil [25]. Moreover, it was recently demonstrated that ACE oil-enriched diet exerts anti-inflammatory, antioxidant and retinoprotective effects in a mouse model of arterial hypertension [26,27,28]. It is worth noting that ACE oil is safe and well tolerated, and no adverse effects have been reported thus far. Thus, we decided to test whether ACE oil could reduce some of the glaucoma-related detrimental effects that result in loss of RGC function and eventually increase RGC resilience to stress conditions. To this aim, we fed mice either an ACE oil-enriched diet or a regular diet and then induced IOP elevation in the animals through intraocular injection of methylcellulose (MCE). MCE is a viscoelastic substance that, when injected into the anterior chamber of the eye, accumulates in the trabecular meshwork and Schlemm’s canal, mechanically blocking aqueous humor outflow [29]. The MCE model was first developed in rabbits [30] and then successfully applied in rats [31] and mice [32]. The retinoprotective effects of the ACE oil-enriched diet supplementation in this glaucoma model were evaluated by assessing whether high IOP-associated glia reactivity and microglia activation, inflammatory processes, oxidative stress, ischemia, apoptosis, RGC loss and electrophysiological properties were affected by the diet.

## 2. Materials and Methods

### 2.1. Animals 

The number of C57BL/6 mice used in the present work, as well as their suffering, was limited according to the 3Rs principles for the ethical usage of animals in scientific research. Twenty-four male mice aged 8–12 weeks were supplied by the Centre for Animal Production and Experimentation (University of Seville, Spain). Of these, twelve were used as controls (six fed regular diet and simply referred to as controls; six fed the ACE oil-enriched diet), while twelve were used as a model of intraocular hypertension (six MCE-injected and fed regular diet; six MCE-injected and fed ACE oil-enriched diet). Mice were housed under standard laboratory conditions of 12 h cycles of light and dark, 23 ± 1 °C, taking food and water ad libitum. Dietary supplementation with the ACE oil-enriched diet was maintained for 6 weeks in the corresponding groups.

### 2.2. Dietary Supplementation

A commercial rodent chow (ROD14IRR, Sodispan Research, Altromin, Germany) was crushed in powder form and then homogeneously mixed with ACE oil to reach a final concentration of 12% (*w*/*w*) of this oil in accordance with previous literature [26,27,28]. The so-prepared oil-powder pellets were maintained at 4 °C in the dark until daily usage.

### 2.3. Weight, Water and Food Intake

Mouse weight was monitored on a weekly basis by placing the animals on a common laboratory balance. Food intake was measured daily by weighing the given and remaining food in each cage. Water intake was monitored on a weekly basis by measuring the given and remaining volumes in the water bottles of each cage.

### 2.4. MCE Injection

MCE (Sigma, Readington, NJ, USA) was prepared as 2% solution in sterile water according to the manufacturer’s instructions. Mice were subjected to anesthesia through inhalation of 3% isoflurane and intracameral injections with 5 μL of MCE were then performed in both the right and the left anterior chambers of each eye, as previously described [32]. 

### 2.5. IOP Measurement 

Rebound tonometry was used to measure IOP through the Icare TonoLab instrument (Icare Finland Oy, Helsinki, Finland), as previously described in different glaucoma mouse models [32,33]. Rebound tonometers can be used either in anesthetized or conscious rodents and exploit a very lightweight magnetic probe that is propelled towards the cornea. The deceleration of the probe after impact correlates with IOP [34]. Special care was taken to align the tonometer tip perpendicular to the center of the cornea while obtaining the measurements. Measurements were performed on awake animals. During the measurements, attention was paid not to stress the mice or to exert pressure on the periocular area. Each measurement with the TonoLab instrument consists of six consecutive readings and the mean of them is displayed as the final result by the device. Different measurements were taken for each eye and then the mean was calculated. Measurements were taken starting from 25 days before the MCE injection and until 12 days after the injection, always between 9 and 12 am.

### 2.6. Measurement of Scotopic and Photopic Electroretinogram (ERG)

Among each experimental group, 4 mice out of 6 were randomly chosen and subjected to electroretinographic recordings.

Full-field ERGs were recorded in four randomly chosen mice in each group. Recordings were carried out using silver–silver chloride corneal electrodes after overnight adaptation in the dark and a Ganzfeld stimulator (CSO, Firenze, Italy). Mice were anesthetized with an intraperitoneal injection of avertin. A needle electrode subcutaneously inserted in the frontal region was used as a reference, while a needle electrode subcutaneously inserted at the base of the tail was used as a ground. Retinal responses were collected simultaneously from both eyes using a data acquisition device (Retimax Advanced; CSO). Recordings were initially taken in the absence of stimuli to measure the background noise level. Light stimuli were calibrated as luminance energy units in candela seconds per meter squared (cd-s/m^2^). The scotopic responses, mainly reflecting rod function, were elicited through a 1.00 log cd-s/m^2^ stimulus, averaging 5 different ERG responses obtained with an interval of 20 s between light flashes. After the acquisition of the scotopic responses, the animals were light-adapted for 30 s before recording photopic cone-mediated responses using a 3 cd-s/m^2^ stimulus on a 30 cd-s/m^2^ rod-saturating background light. For each mouse, 10 waveforms were recorded with an interstimulus interval of 3 s and averaged. In the photopic ERG, the photopic negative response (PhNR) was identified as the first negative deflection after the b-wave, calculating its amplitude relative to the baseline (0 μV). Scotopic and photopic ERG recordings were performed on both eyes simultaneously.

### 2.7. Measurement of Pattern ERG (PERG)

Immediately after scotopic and photopic ERG, the mice were subjected to PERG recordings. PERG responses, mainly generated by RGCs [35], were evoked in anesthetized animals using an alternating pattern of black and white horizontal bars delivered on a stimulus display unit from a commercially available PERG system (Retimax Advanced; CSO). Stimuli consisted of 0.05 cycles/deg black and white bars reversing at 1 Hz, presented at 98% contrast. The pattern stimuli were administered through a light-emitting diode display with a mean luminance of 50 cd/m^2^ aligned at about 20 cm from the corneal surface. A total of 200 signals were averaged. The PERG response was evaluated by measuring the amplitude of the N35–P50 and P50–N95 waves (from the trough of the negative peak, N35, to the peak of the positive peak, P50, and from the peak of the positive peak, P50, to the trough of the negative peak, N95, respectively). The implicit time was determined by measuring the time from the onset of the stimulus to the P50 and N95 peaks. Recordings were taken from the left eyes.

### 2.8. Retina Dissection

Mice were sacrificed by cervical dislocation. Both eyes were immediately enucleated. Retinas were carefully dissected from enucleated eyes under a binocular stereoscopic microscope and maintained at −80 °C until usage for molecular analysis.

### 2.9. RNA Extraction and Quantitative Real-Time PCR (qRT-PCR)

Total RNA was isolated using the RNeasy Micro Kit (Qiagen, Hilden, Germany). Briefly, retinas were homogenized in 150 µL RLT buffer added with β-mercaptoethanol. Homogenates were then subjected to sonication and centrifuged following the manufacturer’s instructions. Supernatants were added with 70% ethanol, centrifuged in the RNeasy Mini spin columns and washed with the supplied buffers. The RNA was eluted in 30 µL RNase-free water. The quantity and purity of RNA were evaluated using the instrument Biophotometer D30 (Eppendorf, Hamburg, Germany). RNA samples were tested for the absence of genomic DNA, and then cDNA synthesis was performed using QuantiTect^®^ Reverse Transcription Kit (Qiagen). qRT-PCR analyses were performed using the SsoAdvanced Univ SYBR Grn Suprmix (Bio-Rad, Hercules, CA, USA) and relative quantification was performed using the comparative 2^–ΔΔCt^ method [36]. Forward and reverse primer sequences are reported in Table 1. The mRNA expression levels were normalized to ribosomal protein L13a (*RPL13a*) as an endogenous control.

### 2.10. Protein Extraction and Western Blot

Retinas were homogenized in RIPA buffer containing dithiothreitol, phosphatase inhibitors and proteinase inhibitor cocktails. Homogenates were then subjected to sonication and centrifuged for 10 min at 10,000× *g*. Protein concentration was determined by the Bradford method. Protein aliquots (40 µg each) were subjected to sodium dodecyl sulfate polyacrylamide gel electrophoresis. The proteins were then transferred onto nitrocellulose membranes. Membranes were blocked at room temperature with 5% non-fat dried milk and then incubated overnight at gentle shaking at 4 °C with primary antibodies. β-actin was used as a housekeeping control. Subsequently, the blots were incubated at room temperature with anti-mouse or anti-rabbit peroxidase-labeled secondary antibody. The signal was revealed with the enhanced chemiluminescence reagent through the GE Healthcare Amersham Imager 600 instrument. Band optical density was evaluated using Image Lab software 6.0.1 (Bio-Rad, Hercules, CA, USA). Data were normalized to β-actin. Antibodies’ references and dilutions are reported in Table 2.

### 2.11. Statistical Analyses

Data are expressed as the mean ± standard error of the mean (SEM). Statistical significance was evaluated using either two-way ANOVA (regarding IOP and weight analyses) or one-way ANOVA (regarding qRT-PCR and Western blot analyses), followed by post-hoc Tukey’s multiple comparisons test. GraphPad Prism 6 software was used to analyze the data. *p* < 0.05 values were considered to be statistically significant.

## 3. Results

### 3.1. ACE Oil-Enriched Diet Does Not Affect Food and Water Intake or Animals’ Weight

**Figure 1:**
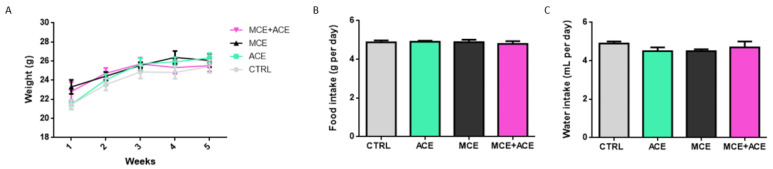
Effects of dietary supplementation on animals’ weight, food and water intake. Animals’ weight (**A**) was monitored weekly, while food intake (**B**) and water intake (**C**) were monitored daily. The weight was not different among the experimental groups represented by control mice (CTRL), non-injected mice fed ACE oil-enriched diet (ACE), mice intraocularly injected with MCE fed regular diet (MCE), and mice intraocularly injected with MCE and fed ACE oil-enriched diet (MCE + ACE). No differences were reported in food or water intake. In (**A**), data are shown as weight means ± SEM (*n* = 6 for each group). Grey circles and line: CTRL; green squares and line: ACE; black triangles and line: MCE; pink triangle and line: MCE + ACE. Statistical Test: Two-way ANOVA. In (**B**,**C**), data are shown as food (g per day) or water (mL per day) intake means of the groups ± SEM (*n* = 6 for each group). Statistical Test: One-way ANOVA.

No variation in animals’ weight, food or water intake was reported among the four experimental groups. The data are shown in Figure 1.

### 3.2. ACE Oil-Enriched Diet Does Not Prevent IOP Elevation

**Figure 2:**
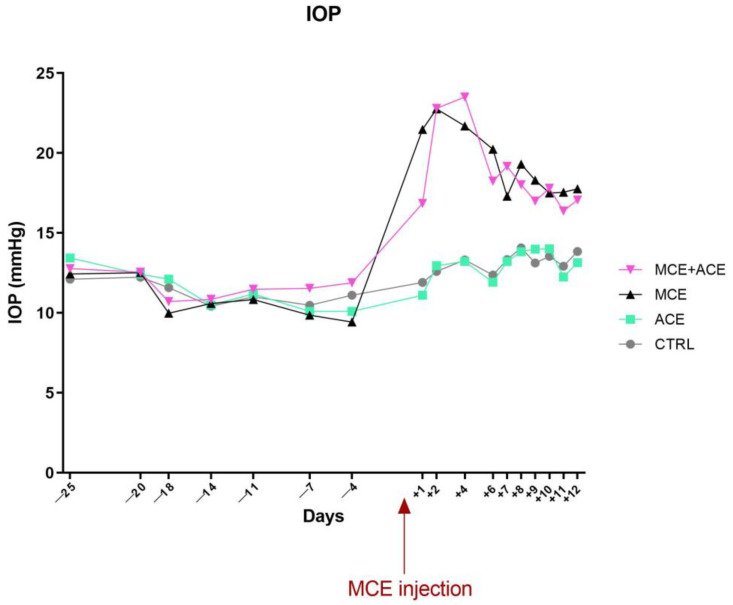
IOP is not affected by an ACE oil-enriched diet. The injection of MCE into the anterior chamber occurred at day 0 (arrow). Neither regular nor ACE oil-enriched diet affected IOP in glaucomatous mice. Data are shown as mean ± SEM (*n* = 6 for each group). Grey circles and line: CTRL; green squares and line: ACE; black triangles and line: MCE; pink triangle and line: MCE + ACE. Statistical significance was evaluated through two-way ANOVA followed by Tukey’s post-hoc multiple comparison test.

As depicted in Figure 2, the ACE oil-enriched diet did not affect IOP compared to animals fed a normal diet. On the other hand, MCE intraocular injection drastically increased IOP *(p* < 0.05), in line with previous results obtained in mice [32,37] and in other animal models [30,31,38,39]. The IOP increase in MCE-injected mice was only slightly attenuated by the ACE oil-enriched diet in the first 24 h after the injection (*p* < 0.0001), but this difference was not preserved afterwards.

### 3.3. ACE Oil-Enriched Diet Suppresses Müller Cells and Microglia Activation in the Glaucomatous Retina

**Figure 3:**
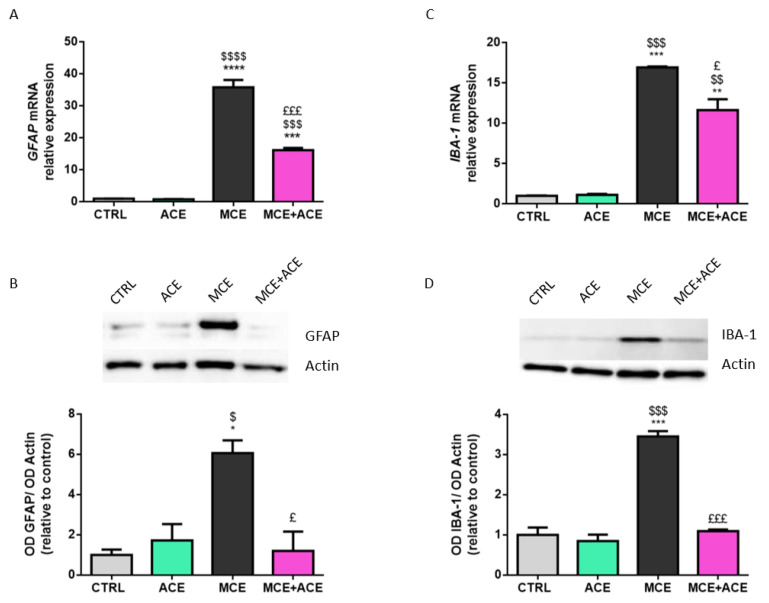
Effects of ACE oil-enriched diet on glia activation. (**A**,**C**) Retinal mRNA expression levels of glial fibrillary acidic protein (GFAP; (**A**)) and ionized calcium-binding adapter molecule 1 (IBA-1; (**C**)) from CTRL, ACE, MCE and MCE + ACE mice. (**B**,**D**) Representative Western blots showing immunoreactive bands and optical density analysis of GFAP (**B**) or IBA-1 (**D**) protein levels in the same groups. The values reported in the mRNA graphs represent the *GFAP* and *IBA-1* mean values of relative expression versus ribosomal protein L13a (RPL13a) ones, ± SEM (*n* = 3). The values reported in the protein graphs represent the GFAP and IBA-1 bands’ optical density mean values normalized versus β-actin ones, ±SEM (*n* = 3). Statistical significance was evaluated through one-way ANOVA followed by post-hoc Tukey’s multiple comparisons test. * *p* < 0.05, ** *p* < 0.01, *** *p* < 0.001 and **** *p* < 0.0001 versus CTRL; ^$^ *p* < 0.05, ^$$^ *p* < 0.01, ^$$$^ *p* < 0.001 and ^$$$$^ *p* < 0.0001 versus ACE; ^£^ *p* < 0.05 and ^£££^ *p* < 0.001 versus MCE.

We evaluated glial fibrillary acidic protein (GFAP) and ionized calcium-binding adapter molecule 1 (IBA1) expression levels within animals’ retinas to assess Müller glia reactivity and microglia activation. The results are shown in Figure 3. Intraocular injection of MCE dramatically increased GFAP and IBA-1 mRNA and protein expression compared to non-injected animals. Notably, ACE oil-enriched diet suppressed Müller cells and microglia activation in the MCE glaucoma model. Indeed, although *GFAP* and *IBA-1* mRNA expression in glaucomatous animals fed an ACE oil-enriched diet remained higher compared to controls, their level was significantly lowered compared to MCE-injected animals fed a normal diet. Moreover, GFAP and IBA-1 protein expression levels returned to a control-like situation in MCE-injected mice fed an ACE oil-enriched diet.

### 3.4. ACE Oil-Enriched Diet Efficiently Reduces Glaucoma-Related Inflammation in the Glaucomatous Retina

**Figure 4:**
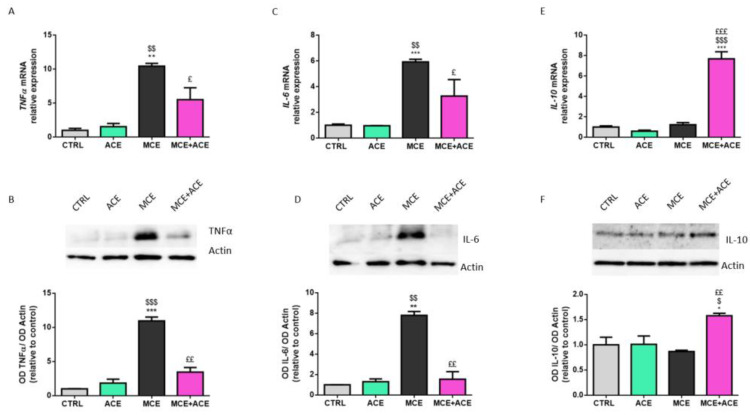
Effects of ACE oil-enriched diet on the expression of inflammatory cytokines. (**A**,**C**,**E**) Retinal mRNA expression levels of tumor necrosis factor-alpha (TNF-α) (**A**), interleukin 6 (IL-6) (**C**) and interleukin 10 (IL-10) (**E**) were evaluated in retinae from CTRL ACE, MCE and MCE + ACE mice. The values reported in the mRNA graphs represent the *TNF-α*, *IL-6* and *IL-10* mean values of relative expression versus *RPL13a* ones, ± SEM (*n* = 3). (**B**,**D**,**F**) Representative Western blots showing immunoreactive bands and optical density analysis of TNF-α (**B**), IL-6 (**D**) or IL-10 (**F**) protein levels in the same groups. The values reported in the protein graphs represent the TNF-α, IL-6 and IL-10 band’s optical density mean values normalized versus β-actin ones, ± SEM (*n* = 3). Statistical significance was evaluated through one-way ANOVA followed by post-hoc Tukey’s multiple comparisons test. * *p* < 0.05, ** *p* < 0.01 and *** *p* < 0.001 versus CTRL; ^$^ *p* < 0.05, ^$$^ *p* < 0.01 and ^$$$^ *p* < 0.001 versus ACE; ^£^ *p* < 0.05, ^££^ *p* < 0.01 and ^£££^ *p* < 0.001 versus MCE.

Due to the well-demonstrated beneficial properties of ACE oil [26,27,28], we investigated the potential anti-inflammatory effect of an ACE oil-enriched diet in the MCE glaucoma model. The results reported in Figure 4A–D show that the mRNA and protein expression of the pro-inflammatory cytokines tumor necrosis factor alpha (TNFα) and interleukin 6 (IL-6) were drastically increased in the MCE model, while the ACE oil-enriched diet prevented this upregulation, restoring a control-like situation in both IL-6 and TNFα mRNA and protein expression. On the other hand, the mRNA and protein levels of the anti-inflammatory cytokine interleukin-10 (IL-10) were not affected by the MCE injection (Figure 4E,F). Conversely, the ACE oil-enriched diet significantly increased the mRNA and protein expression of IL-10 in MCE-injected animals.

### 3.5. ACE Oil-Enriched Diet Restored a Control-like Expression of Oxidative Stress Markers in the Glaucomatous Retina

**Figure 5:**
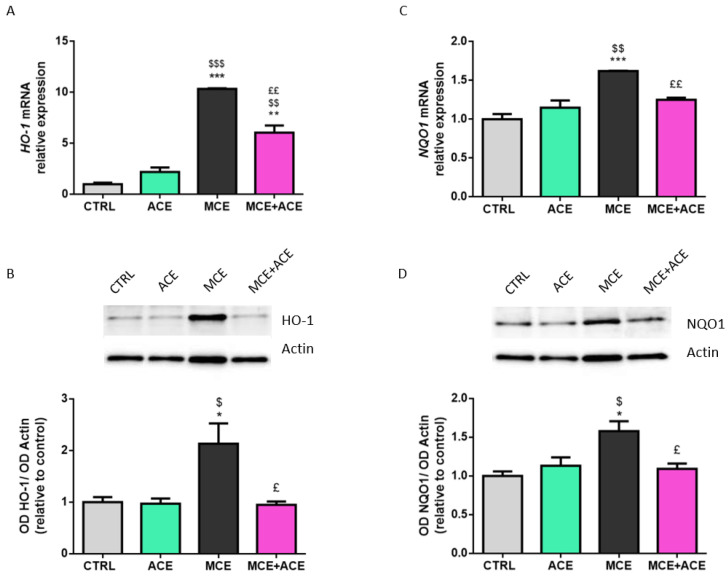
(**A**,**C**) Retinal mRNA expression levels of hemeoxygenase-1 (HO-1) (**A**) and nicotinamide adenine dinucleotide phosphate quinine oxidoreductase 1 (NQO1) (**B**) were evaluated in retinae from CTRL ACE, MCE and MCE + ACE mice. The values reported in the mRNA graphs represent the *HO-1* and *NQO1* mean values of relative expression versus *RPL13a* ones, ± SEM (*n* = 3). (**B**,**D**) Representative Western blots showing immunoreactive bands and optical density analysis of HO-1 (**B**) or NQO1 (**D**) protein levels in the same groups. The values reported in the protein graphs represent the HO-1 and NQO1 bands’ optical density mean values normalized versus β-actin ones, ± SEM (*n* = 3). Statistical significance was evaluated through one-way ANOVA followed by post-hoc Tukey’s multiple comparisons test. * *p* < 0.05, ** *p* < 0.01 and *** *p* < 0.001 versus CTRL; ^$^ *p* < 0.05, ^$$^ *p* < 0.01 and ^$$$^ *p* < 0.001 versus ACE; ^£^ *p* < 0.05 and ^££^ *p* < 0.01 versus MCE.

To evaluate the redox status of animals’ retinas, we analyzed the expression of some biological markers of oxidative stress, such as hemeoxygenase-1 (HO-1) and nicotinamide adenine dinucleotide phosphate quinine oxidoreductase 1 (NQO1). As reported in Figure 5, HO-1 and NQO1 expression increased in MCE-injected animals, both at the mRNA and protein levels. *HO-1* mRNA remained upregulated also in MCE-injected mice fed ACE oil-enriched diet, although its expression was significantly lower compared to glaucomatous animals fed a normal diet. On the other hand, HO-1 protein level and NQO1 mRNA and protein levels were decreased to control levels in glaucomatous animals fed an ACE oil-enriched diet.

### 3.6. ACE Oil-Enriched Diet Alleviates Ischemia in the Glaucomatous Retina

**Figure 6:**
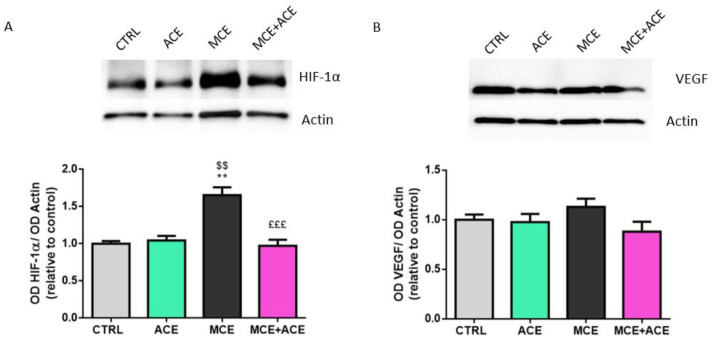
An ACE oil-enriched diet ameliorates ischemia. (**A**,**B**) Representative Western blots showing immunoreactive bands and optical density analysis of hypoxia-inducible factor 1 (HIF-1α) (**A**) and vascular endothelial growth factor (VEGF) (**B**) protein levels in retinae from CTRL ACE, MCE and MCE + ACE mice. The values reported in the protein graphs represent the HIF-1α and VEGF band’s optical density mean values normalized versus β-actin ones, ± SEM (*n* = 3). Statistical significance was evaluated through one-way ANOVA followed by post-hoc Tukey’s multiple comparisons test. ** *p* < 0.01 versus CTRL; ^$$^ *p* < 0.01 versus ACE; ^£££^ *p* < 0.001 versus MCE.

To evaluate whether an ACE oil-enriched diet could affect the ischemic injury caused by glaucoma [40], the α subunit of hypoxia-inducible factor 1 (HIF-1α) and the vascular endothelial growth factor (VEGF) protein expression levels were evaluated through Western blotting. As shown in Figure 6A, the glaucoma-related overexpression of HIF-1α observed in MCE-injected animals fed a normal diet was completely suppressed by ACE oil-enriched diet. Indeed, MCE-injected animals fed an ACE oil-enriched diet showed a control-like protein expression level of HIF-1α. Neither MCE injection nor the ACE oil-enriched diet affected VEGF expression levels (Figure 6B).

### 3.7. ACE Oil-Enriched Diet Prevents Apoptosis in the Glaucomatous Retina

**Figure 7:**
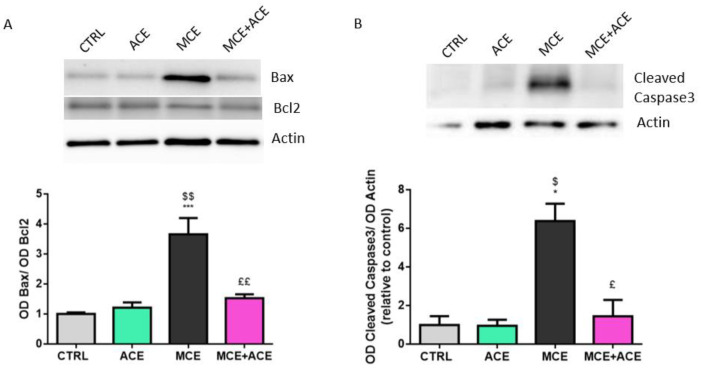
ACE oil-enriched diet prevents apoptosis within the retina. (**A**,**B**) Representative Western blots showing immunoreactive bands and optical density analysis of B-Cell Lymphoma 2 (Bcl2) associated X protein (Bax)/Bcl2 ratio (**A**) and cleaved caspase 3 (**B**) protein levels in retinae from CTRL ACE, MCE and MCE + ACE mice. The values reported in the protein graphs represent the Bax/Bcl2 ratio and cleaved caspase 3 bands’ optical density mean values normalized versus β-actin ones, ± SEM (*n* = 3). Statistical significance was evaluated through one-way ANOVA followed by post-hoc Tukey’s multiple comparisons test. * *p* < 0.05, *** *p* < 0.001 versus CTRL; ^$^ *p* < 0.05 and ^$$^ *p* < 0.01 versus ACE; ^£^ *p* < 0.05 and ^££^ *p* < 0.01 versus MCE.

In order to assess whether an ACE oil-enriched diet could affect the apoptosis rate that characterizes glaucoma, we evaluated the protein expression levels of three different species involved in the apoptosis process (Figure 7). Within the retinas of glaucomatous mice, we observed a marked increase in the B-Cell Lymphoma 2 (Bcl2) associated X protein (Bax)/Bcl2 ratio. Particularly, we reported enhanced protein levels of the pro-apoptotic Bax and unaltered levels of the anti-apoptotic Bcl2. In line with this, levels of the cleaved (and, therefore, active) form of caspase 3 were also increased in MCE-injected animals. Notably, ACE oil was able to decrease Bax/Bcl2 ratio and to inhibit caspase 3 activation. No significant difference in Bax/Bcl2 ratio or in cleaved caspase 3 protein level was reported between control mice and MCE-injected animals fed ACE oil-enriched diet.

### 3.8. ACE Oil-Enriched Diet Restores Retina Electrophysiological Properties

Finally, retinal function was assessed by measuring the amplitude of different components in scotopic or photopic full-field ERG and in PERG, as well as the implicit time of the PERG wave. The amplitude of the a- and b-waves in the scotopic ERG as well as the amplitude of the b-wave in the photopic full-field ERG were used to evaluate outer and mid-retinal function, while the amplitude of the PhNR measured in the photopic b-wave was used to evaluate inner retinal function [41]. Instead, PERG responses were used to evaluate RGC function [35]. Scotopic and photopic ERG are considered insensitive to glaucoma and used to verify that the procedure to increase IOP has not induced generalized retinal damage in the experimental models [42]. However, alteration of the PhNR in the photopic ERG can occur in glaucoma, as this negative wave is generated by the activity of peripheral RGCs [43].

**Figure 8:**
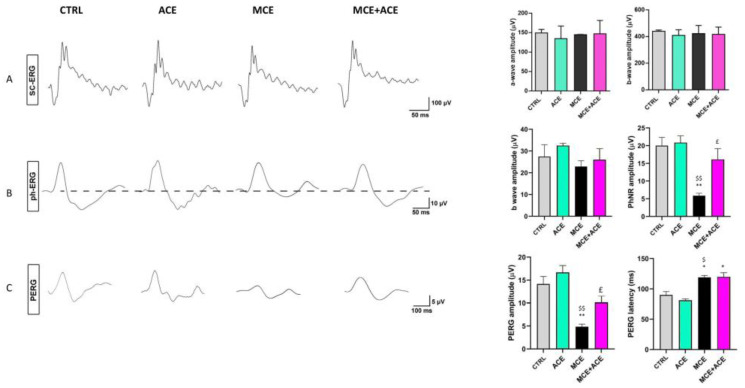
The effects of dietary supplementation on retina functionality were evaluated by scotopic, photopic full field electroretinogram (ERG) and pattern ERG (PERG). ERG recordings were carried out in CTRL, ACE, MCE and MCE + ACE mice. (**A**) Representative scotopic ERG traces with a- and b- waves, whose amplitudes were evaluated as shown by the graphs on the right. (**B**) Representative photopic ERG traces, showing b-waves with photopic negative response (PhNR), whose amplitudes were evaluated as shown by the graphs on the right. (**C**) Representative PERG traces showing the two negative peaks (N35 and N95) and the positive peak P50; PERG amplitude and latency were evaluated as shown by the graphs on the right. Mean amplitudes of ERG responses were evaluated as changes from baseline, normalized to the amplitude measured in the control animals. MCE did not affect the amplitude of the scotopic a-wave, scotopic b-wave and photopic b-wave, while it reduced the amplitude of PhNR. Dietary supplementation partially prevented a reduction in PhNR amplitude. The PERG amplitude was reduced by MCE, an effect that was prevented by dietary supplementation. The mean implicit time of PERG was increased by MCE, an effect that was not abolished by dietary supplementation. Data are shown as the mean of the groups ± SEM (*n* = 4). Statistical significance was evaluated through one-way ANOVA followed by the Newman–Keuls multiple comparison post-hoc test. * *p* < 0.05 and ** *p* < 0.01 versus CTRL; ^£^ *p* < 0.05 versus MCE. ^$^ *p* < 0.05 and ^$$^ *p* < 0.01 versus ACE.

As shown in Figure 8, we found no differences in scotopic a- and b-waves or photopic b-waves among the experimental groups. On the other hand, both photopic PhNR and PERG amplitudes were reduced following the MCE injection, in line with previous reports [32,44,45,46,47]. Moreover, PERG latency was increased in the MCE-injected animals. Importantly, in glaucomatous animals fed an ACE oil-enriched diet, the special diet was able to preserve the amplitude of both PhNR and PERG, although it was not effective in restoring PERG latency.

## 4. Discussion

The present study provides evidence about the efficacy of an ACE oil-enriched diet in a mouse model of hypertensive glaucoma. In particular, the present findings demonstrate that although no significant effects on IOP elevation could be obtained following the treatment, the diet was able to suppress Müller glia reactivity and microglia activation as likely result of ACE anti-inflammatory properties. Moreover, the altered redox balance and ischemic status typical of ocular hypertensive conditions were restored to a control-like situation by the ACE oil-enriched diet. Both the anti-inflammatory and antioxidant properties of ACE correlated with a significant preservation of retinal cell viability and function, as demonstrated by the inhibition of MCE-induced apoptotic activity and the preservation of RGC-related ERG parameters, respectively.

Although the precise mechanism underlying glaucoma-related neurodegeneration remains to be further elucidated, it is well-known that glia activation, neuro-inflammation, oxidative stress, and ischemia worsen the RGC death rate following optic nerve damage [48]. As in many neurodegenerative pathologies, dysfunctions in glaucoma mouse models are associated with reactive gliosis and microglia activation, usually measured by increases in GFAP [49] and IBA-1 [50,51] expression levels, respectively. Consistently, GFAP and IBA-1 were strikingly upregulated in MCE-injected mice compared to the control. Triggering of gliosis in Müller cells and activation of microglia were abolished in glaucomatous animals that received the ACE oil-enriched diet, in line with previous data demonstrating that antioxidant/anti-inflammatory interventions reduce gliosis and microglia activation in glaucoma [31,32,37,52,53].

Notably, gliosis level exacerbates along with glaucoma progression [54] and plays an important role in pathological RGC degeneration [55]. Indeed, as a consequence of glaucoma-related harmful stimuli (including optic nerve injury, IOP elevation, and excitotoxicity), reactive glial cells redistribute throughout the retina and the optic nerve, where they start producing inflammation mediators such as IL-6 and TNF-α [56]. Glaucoma-related alterations at the optic nerve head are also spatially associated with morphologically amoeboid microglia expressing markers of activation, inflammatory mediators and cytokines, complement molecules, and metalloproteases [57]. In line with this, the present results show that in the MCE glaucoma model, there was an enhanced expression of both IL-6 and TNF-α, in accordance with previous findings [31,32,58]. Conversely, unlike previous data obtained in MCE rodent models [31,32], no change in the protein expression level of the anti-inflammatory cytokine IL-10 was detected after glaucoma induction. However, taken together, the current results suggest the presence of an elevated inflammation level within the glaucomatous retinas of MCE-injected animals compared to the control.

The aim of activation of the inflammatory process is to counteract an insult and its damaging effects, thus exerting a protective function in the early phase of pathologies, such as glaucoma. Nevertheless, when the noxious stimulus is severe and/or prolonged over time, the balance between the beneficial and harmful effects of the inflammatory response becomes impaired. This leads to deleterious outcomes with implications in both induction and progression of pathologies. Particularly in glaucoma, this results in detrimental consequences on RGC survival, thus promoting neurodegeneration [56]. The several ways in which TNF-α affects RGC viability in glaucoma are well known and have been recently revised by Baudouin et al. [59]. On the other hand, IL-6 expression has been previously correlated with neuroprotective, regenerative and proliferative responses in different glaucoma models [60,61,62,63]. However, more recently, in a microbead-induced glaucoma murine model, it was shown that IL-6 plays a key role in the process of structural degeneration of the optic nerve and that its absence prevents not only this latter, but also vision loss [64]. As shown by the present results, the inflammatory milieu of glaucomatous animals was restored to physiological conditions by an ACE oil-enriched diet. Moreover, in MCE-injected animals, an ACE oil-enriched diet upregulated the expression of IL-10, suggesting the triggering of some protective mechanism in response to glaucoma induction. These results are in line with previous data reporting that nutritional support with natural anti-inflammatory compounds is able to prevent inflammatory triggers in glaucoma models. For instance, spearmint, a plant-based polyphenolic extract, reduces oxidative stress and inflammation and improves retina functions and RGC survival in an MCE-induced glaucoma rat model [39]. Another promising natural compound for counteracting glaucoma is represented by green tea catechins, which show strong anti-oxidative and anti-inflammatory properties [65].

Gliosis and inflammation are not the only players involved in RGC degeneration. Notably, the triad “inflammation, oxidative stress, ischemia/hypoxia” represents a vicious circle where any element can trigger the activation of the others and sustain the neurodegenerative processes over time [66]. In particular, oxidative stress, a major risk factor in human glaucoma, crucially affects RGC resilience and contributes to their apoptotic death [67]. Indeed, oxidative stress alters the aqueous humor outflow pathway by causing trabecular meshwork degeneration [68] and contributes to optic nerve atrophy and optic disc cupping enlargement [69]. We report an increased antioxidant response in the MCE group, based on HO-1 and NQO1 expression levels, in accordance with recent data [70]. The overexpression of these antioxidant enzymes in MCE-injected animals was almost completely prevented by an ACE oil-enriched diet. The finding that there is an apparent discrepancy in HO-1 mRNA and protein levels could be due to intense enzyme turnover. Indeed, it has been shown that HO-1 accumulates more into lysosomes of retinal pigment epithelial cells in exudative age-related macular degeneration patients than in the cytoplasm [71]. Thus, the enzyme could be subject to a higher turnover due to degradation induced by lysosomes compared to its own mRNA. The overall action ACE oil exerted on antioxidant enzymes can be explained by assuming that the four weeks of diet prior to MCE injection might have allowed us to buffer the glaucoma-triggered increase in free radical species formation, thus decreasing the upstream oxidative stress-driven expression of HO-1 and NQO1.

A further important contribution to RGC death in glaucoma is ischemic damage. Ischemia is a pathological condition characterized by a reduction of blood perfusion, leading to accumulation of metabolic debris and insufficient nutrients and oxygen supply [72]. Glaucoma patients experience reduced ocular perfusion in both the early and late stages of the pathology; tissue hypoxia in the optic nerve head and/or retina can develop either because of or independently from the elevated IOP and is associated with pathogenic mechanisms underlying optic nerve degeneration [73,74]. Indeed, within the retina, RGCs are the most sensitive and vulnerable cells to ischemic injury, which promotes their death by apoptosis [40]. HIF-1α is a key regulator of the response to ischemic damage [75] and its upregulation was demonstrated in the retina and in the optic nerve head of glaucomatous human donors [74]. Although there is evidence that HIF-1 signaling can exert protective effects, it also contributes to cell death and tissue damage when its activation is prolonged over time [75]. Consistently, suppression of HIF-1α upregulation characterizing a rat model of retinal ischemia exerts a protective action against ischemia itself and reduces retinal apoptosis [76]. Similarly, in the present study, the striking MCE-induced upregulation of HIF-1α was completely suppressed by an ACE oil-enriched diet, suggesting that ACE oil might exert protective functions against retinal ischemic damage characterizing glaucoma. A well-known target of HIF-1α is VEGF, which is deeply involved in both physiological and pathological angiogenesis [77]. We found no significant alteration of VEGF in MCE-injected mice or in glaucomatous animals fed ACE-oil-enriched diet. There is a lack of previous information about VEGF levels within the retina of MCE-injected mice. However, in a rat model of hypertensive glaucoma obtained through hypertonic saline solution injection in the episcleral veins, it was demonstrated that the retinal levels of the proangiogenic VEGF-A164 isoform were not altered compared to control animals five days or ten days after glaucoma induction [78]. Contrarily, in the retina of a laser-burn induced hypertensive glaucoma rat model, VEGF protein levels raised and remained higher compared to the control up to ten weeks after treatment [79]. Therefore, VEGF levels within the retinas of glaucoma animal models might be dependent on the model itself, as well as on the analyzed VEGF isoform. In the plasma of glaucoma patients, VEGF levels are increased compared to healthy individuals [80]; the aqueous humor VEGF levels of glaucoma patients are also increased when compared to their own plasma VEGF levels [81]. This might suggest an involvement of VEGF in glaucoma pathological conditions. Nevertheless, it was recently hypothesized that, in glaucomatous human eyes, VEGF inhibition would play a role in the progressive thinning of the RGC layer [82]. Moreover, it is well known that HIF-1α can undergo post-transcriptional modifications able to affect its activity and thus its potential effect on the expression of its targets, such as VEGF. Indeed, many of these modifications can result in ubiquitination and degradation, therefore affecting its stability or directly modifying its localization/activity [83].

In the present study, MCE-induced IOP elevation, the consequent activation of microglia and gliosis triggering, neuroinflammation, oxidative stress and ischemia are associated with enhanced apoptosis within the retina. Indeed, we observed increased levels of Bax/Bcl2 ratio, where Bax is a critical executioner of mitochondrial-regulated cell death through permeabilization of the mitochondrial outer membrane [84]. On the contrary, Bcl2 exerts an anti-apoptotic function suppressing cytochrome c release by mitochondria [85] and inhibiting Bax via its Bcl-2 homology (BH)1 and BH2 domains [86]. Particularly, Bax was overexpressed in MCE-injected animals and the ACE oil-enriched diet restored control-like expression of this pro-apoptotic protein; on the other hand, we reported no alterations in Bcl2 levels, in accordance with previous results on the same model [32]. Moreover, caspase 3 was remarkably activated in glaucomatous animals. Caspase 3 cleavage marks the beginning of the apoptosis execution phase, where intrinsic and extrinsic apoptosis pathways converge. In this last phase of programmed cell death, execution caspases, the most important of which is caspase 3, activate several endonucleases and proteases that degrade nuclear material and nuclear and cytoskeletal proteins, respectively [85]. As shown here, the ACE oil-enriched diet was able to completely suppress caspase 3 activation. These results suggest a powerful effect of ACE oil on the inhibition of glaucoma-dependent apoptotic processes in the retina.

RGC death is preceded by the loss of function of these cells [87]. Therefore, RGCs, although still alive, may no longer be functional. Thus, we decided to evaluate RGC electrophysiological activity through ERG recording. As shown here, the glaucoma-related reduction of RGC function, as evaluated by the decreased amplitude of PhNR and PERG waves, is reversed by the ACE oil-enriched diet. This is in line with the results obtained in rodent models of glaucoma fed with nutraceuticals with antioxidant/anti-inflammatory properties [32,47,88]. Overall, these results suggest that an ACE oil-enriched diet could induce an almost complete recovery of glaucoma-related retinal dysfunctions, with discrete efficacy in preserving RGC functionality and promoting their resilience.

The protective effects of ACE oil against the deleterious effects of glaucoma could depend on the oil composition, which is quite peculiar. Indeed, ACE oil shows a higher quantity of sterols, tocopherols (e.g., vitamin E) and triterpene acids compared to the classic extra-virgin olive oil [26,89]. In this respect, a recently published work showed that in a mouse model of glaucoma ACE oil have better retinoprotective activity than a reference extra-virgin olive oil in terms of antioxidant, anti-inflammatory and antifibrotic effects, suggesting that the peculiar composition of ACE oil may be responsible for its improved features [28]. Among these components, vitamin E is a powerful fat-soluble antioxidant that is able to prevent the oxidation of polyunsaturated fatty acids in cell membranes. Its deficiency increased the RGC death ratio in a rat model of IOP elevation, leading to higher levels of retinal lipid peroxidation [90]. Moreover, a neuroprotective effect of an oral supplement of α-tocopherol acetate was reported in a nonrandomized placebo-controlled study on 30 glaucomatous patients [91]. Triterpene acids also show anti-inflammatory, antioxidant and neuroprotective properties [92,93]. Their efficacy against glaucoma-related dysfunctions is progressively emerging from the literature in different glaucoma models, such as optic nerve crush [94], trabecular laser photocoagulation [95], and microsphere injection [46]. Moreover, some of them have been traditionally used in oriental medicine over the centuries as a natural remedy against inflammation [95]. Thus, the unique composition of ACE oil can account for its strong anti-inflammatory and antioxidant effects, which, cross-talking with each other, might contribute to the antiapoptotic and neuroprotective outcomes observed in this work.

The ameliorative effects exerted on the glaucomatous retina by an ACE oil-enriched diet do not appear to rely on IOP reduction in the MCE model; similar results were previously obtained in rodent glaucoma models treated with other nutraceutical compounds [32,46,53,95]. Nevertheless, Santana-Garrido et al. recently described the ability of ACE oil-enriched diet to restore IOP levels in Nω-nitro-L-arginine methyl ester (L-NAME)-treated hypertensive mice [28]. L-NAME is able to inhibit nitric oxide synthases, particularly reducing endothelial nitric oxide production and thus inducing systemic vasoconstriction [96]. In this latter case, the increase in IOP is a consequence of systemic hypertension, while in the current model, IOP rises due to the blockage of Schlemm’s canal exerted by MCE. The substantial differences between the two models might explain the different effects of ACE oil dietary supplementation on IOP.

The present results suggesting that ACE oil is able to activate non-IOP-related mechanisms to exert its protective effects could be important since high IOP glaucoma patients often experience no benefits against the progression of the pathology despite the efficacy of IOP lowering therapies [13]. Indeed, besides elevated IOP, there are several recognized contributing factors and many pathological events that lead to RGC degeneration in glaucoma, such as perturbation of neurotrophic signaling, excitotoxicity, trophic factor deprivation, oxidative stress, dysfunction of mitochondria, protein misfolding, inflammation, hypoxic/ischemic injuries, autoimmunity, and autophagy [48,97]. Each of these situations contributes to the progression of the disease and, thus, represents a potential target for neuroprotective, IOP-independent, therapeutic approaches.

## 5. Conclusions

A growing body of evidence is progressively highlighting the efficacy of natural compounds characterized by antioxidant, anti-inflammatory, and anti-apoptotic properties in preventing RGC death in retinal degeneration models, supporting RGC resilience in stress conditions. With the present work, we expanded this knowledge, adding to the list ACE oil as a very promising candidate to counteract glaucoma-related adverse events and preserve RGC functionality. Despite that the putative clinical effectiveness of ACE oil has yet to be demonstrated, what can be extrapolated from these preliminary data is that nutritional supplementation with ACE oil represents an interesting adjuvant in the management of glaucoma.

## Figures and Tables

**Table 1:** List of primers used for Real-Time PCR experiments.

Gene	Forward Primer (5′→3′)	Reverse Primer (5′→3′)
GFAP	AGGGAGTGGAGGAGTCATTCG	CGGAGACGCATCACCTCT
IBA-1	CGAATGCTGGAGAAACTTGG	AGCCCCACCGTGTGACAT
TNF α	GCCTCTTCTCATTCCTGCTTG	CACTTGGTGGTTTGCTACGAC
IL-6	CCAAGAACGATAGTCAATTCCAGA	CATCAGTCCCAAGAAGGCAAC
IL-10	CCAAGCCTTATCGGAAATGA	TTTTCACAGGGGAGAAATCG
HO-1	AAGCCGAGAATGCTGAGTTCA	GCCGTGTAGATATGGTACAAGGA
NQO1	AGGATGGGAGGTACTCGAATC	AGGCGTCCTTCCTTATATGCTA
RPL13a	CACTCTGGAGGAGAAACGGAAGG	GCAGGCATGAGGCAAACAGTC

**Table 2:** List of primary antibodies used for Western blot experiments. SCB = Santa Cruz Biotechnology (Santa Cruz, CA, USA).

Primary Antibody	Origin	Dilution	Secondary Antibody	Dilution	Reference
Anti-GFAP	Mouse monoclonal	1:4000	Goat Anti-Mouse	1:5000	Santa Cruz Biotechnol-ogy (SCB), Dallas, TX, USA
Anti-IBA-1	Rabbit polyclonal	1:1000	Goat Anti-Rabbit	1:5000	Abcam, Cambridge, UK
Anti-TNF α	Mouse monoclonal	1:1000	Goat Anti-Mouse	1:2000	SCB
Anti-IL-6	Mouse monoclonal	1:1000	Goat Anti-Mouse	1:2000	SCB
Anti-IL-10	Mouse monoclonal	1:100	Goat Anti-Mouse	1:1000	SCB
Anti-HO-1	Rabbit poly-clonal	1:1000	Goat Anti-Rabbit	1:5000	Abcam
Anti-NQO1	Rabbit polyclonal	1:500	Goat Anti-Rabbit	1:5000	Abcam
Anti-HIF-1α	Rabbit polyclonal	1:1000	Goat Anti-Rabbit	1:5000	Abcam
Anti-VEGF	Rabbit polyclonal	1:1000	Goat Anti-Rabbit	1:5000	Abcam
Anti-Bax	Rabbit polyclonal	1:500	Goat Anti-Rabbit	1:5000	Abcam
Antii-Bcl2	Rabbit polyclonal	1:500	Goat Anti-Rabbit	1:5000	Abcam
Anti-Cleaved Caspase3	Rabbit polyclonal	1:1000	Goat Anti-Rabbit	1:2000	Cell Signaling, Danvers, MA, USA
Anti-β-actin	Mouse monoclonal	1:20,000	Goat Anti-Mouse	1:30,000	SCB

## Data Availability

Data is contained within the article. The data presented in this study are available on request from the corresponding authors.
